# Supplementary material for: Multiple mechanisms of aminoglycoside ototoxicity are distinguished by subcellular localization of action
Source: Front Neurol. 2024 Nov 14;15:1480435. doi: 10.3389/fneur.2024.1480435 (PMC11602426; doi:10.3389/fneur.2024.1480435)

Supplemental Figure 1

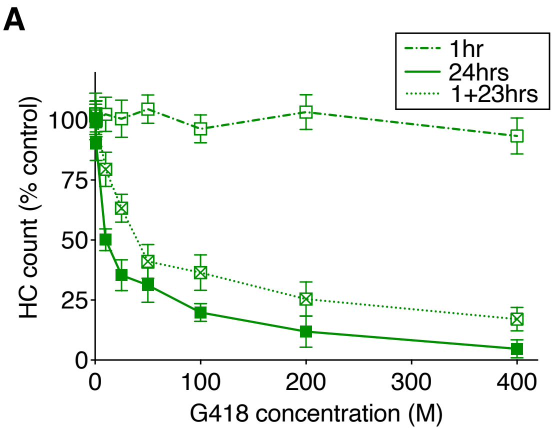

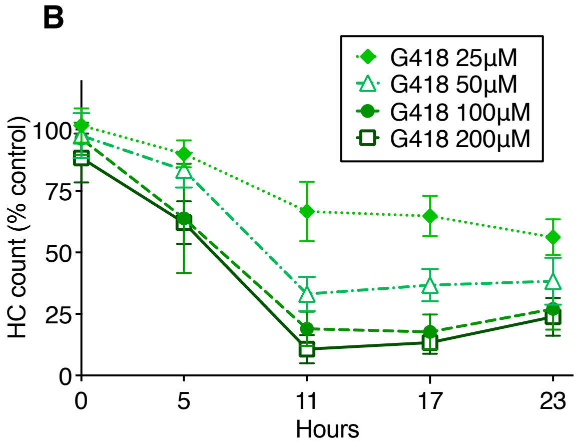

Supplemental Figure 2

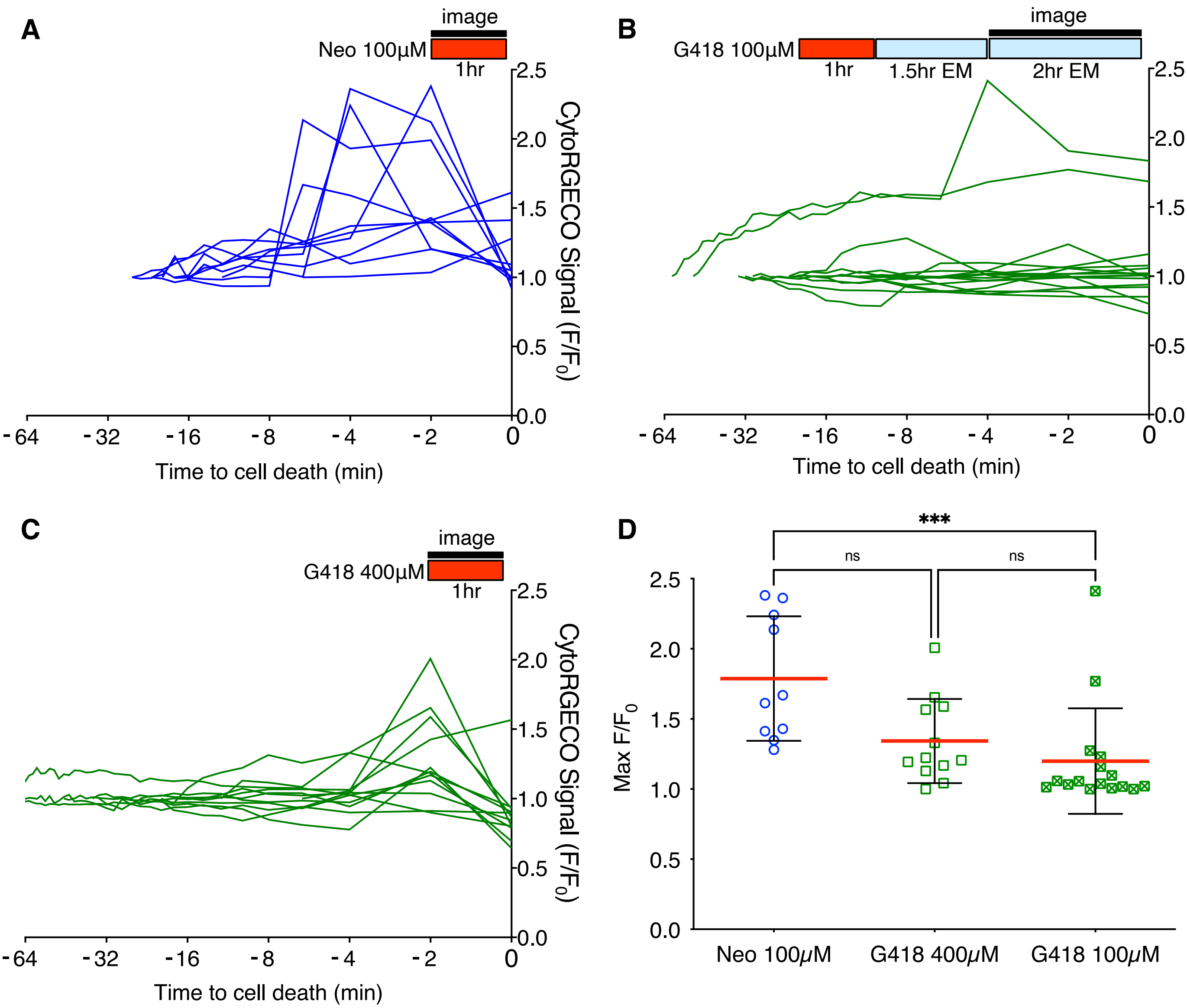

Supplemental Figure 3

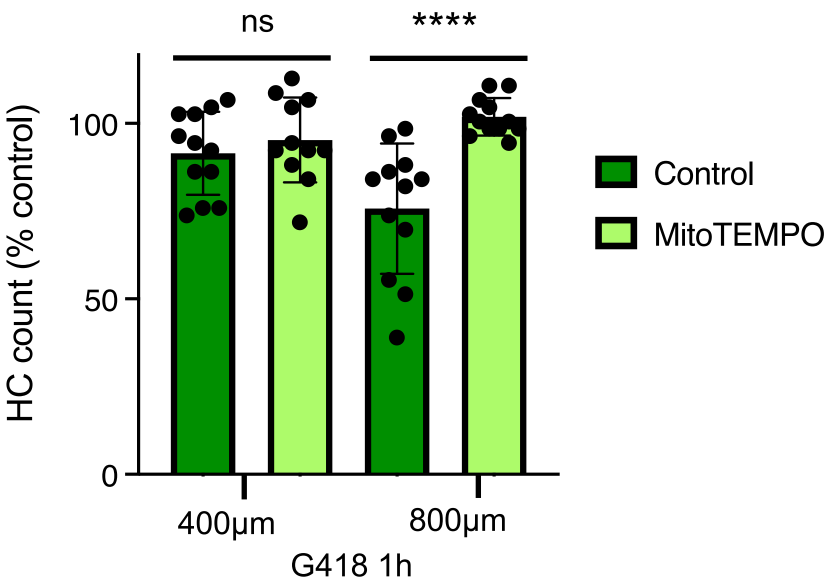

Supplemental Figure 4

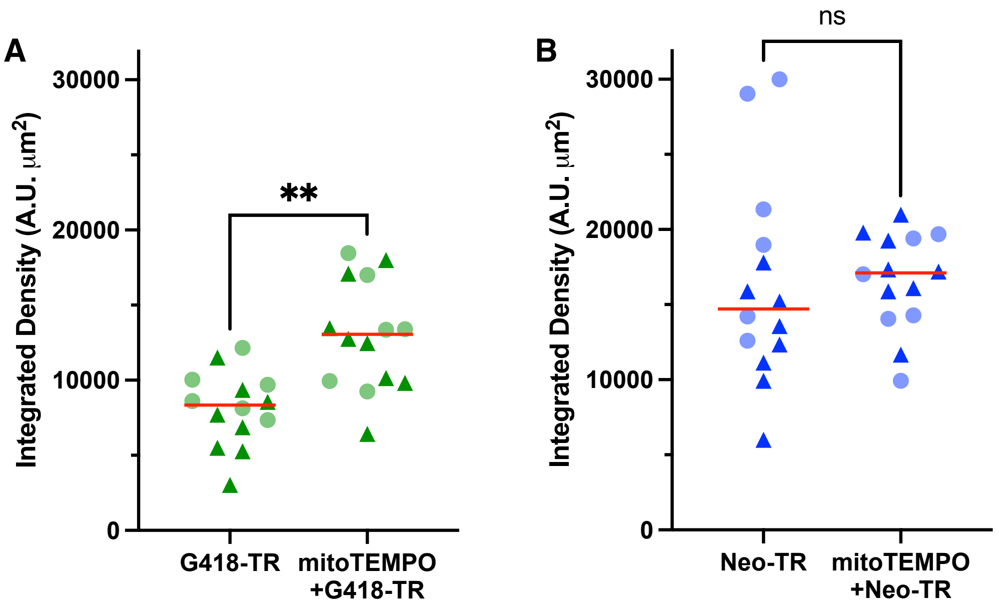

Supplemental Figure 5

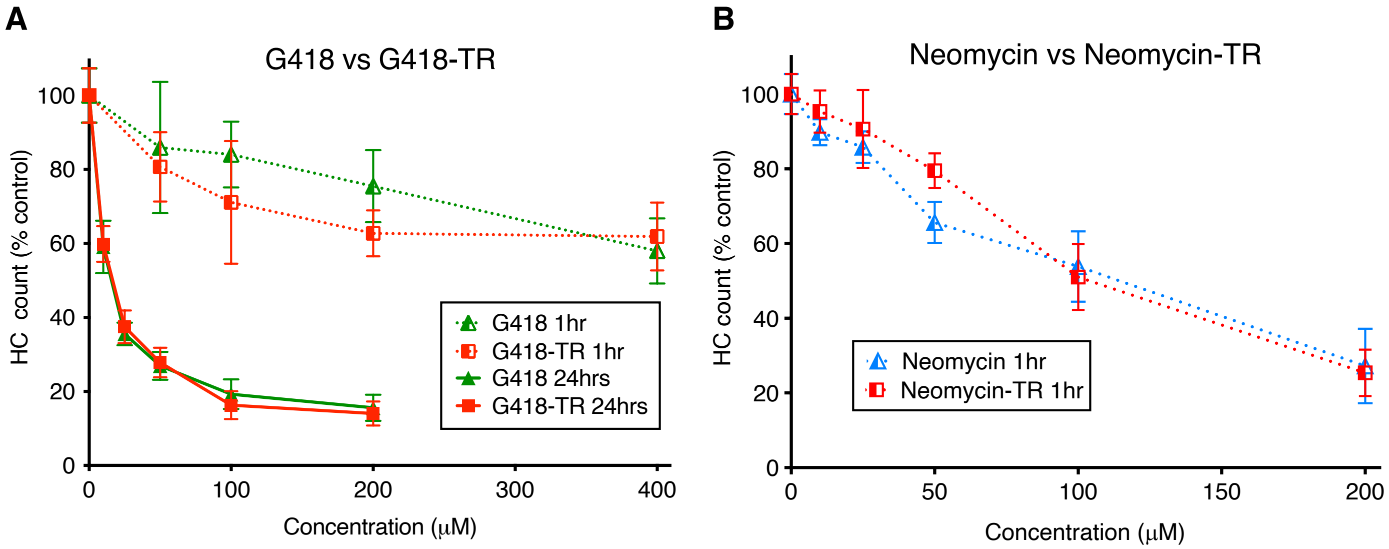

Supplemental Figure 6

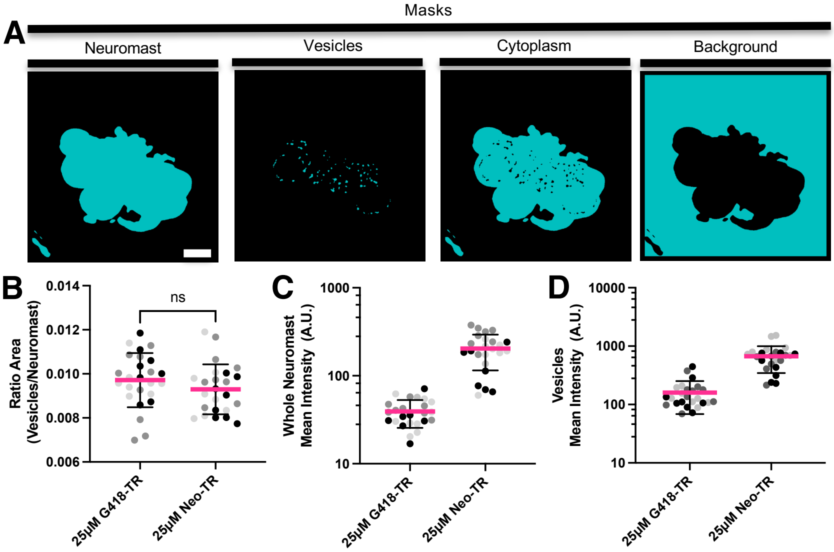

Supplemental
Figure 7

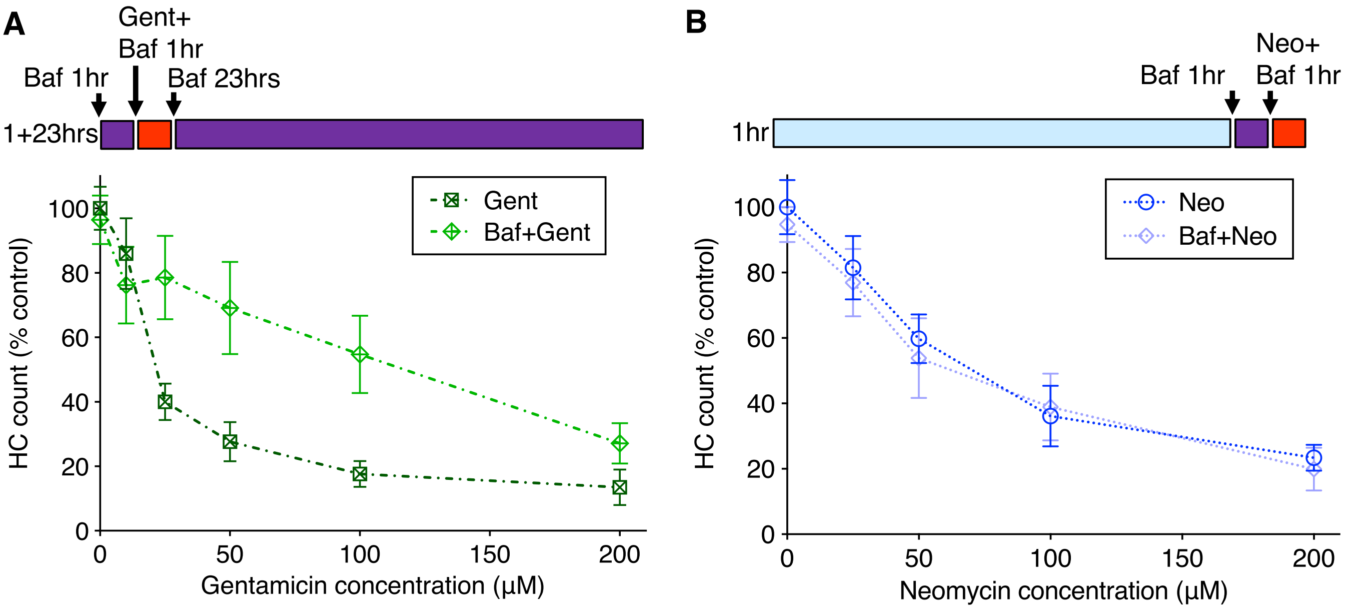

Supplemental Figure 8

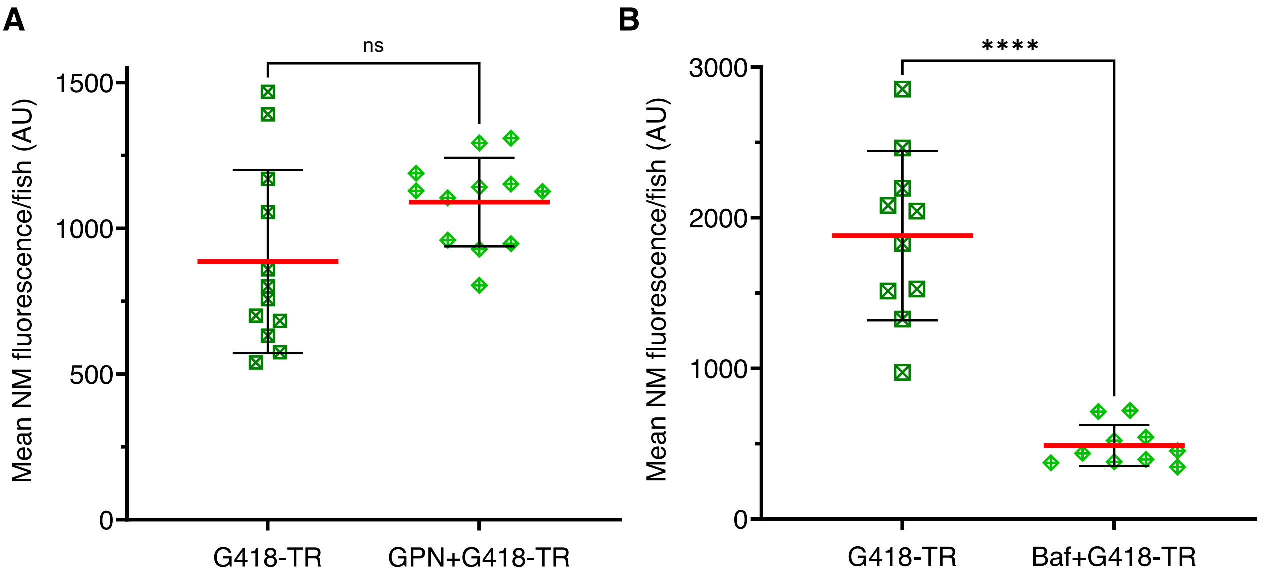

Supplement: Supplementary file 1 [file Data_Sheet_1.pdf]
