# Supplementary material for: Multiple mechanisms of aminoglycoside ototoxicity are distinguished by subcellular localization of action
Source: Front Neurol. 2024 Nov 14;15:1480435. doi: 10.3389/fneur.2024.1480435 (PMC11602426; doi:10.3389/fneur.2024.1480435)
Supplement: Supplementary file 2 [file Table_1.docx]

**SIUPPLEMENTAL INFORMATION**

**Supplemental Figure 1.** A) G418 causes delayed hair cell death comparable to gentamicin. Dose-dependent loss of hair cells after treatment with G418 for 1hr, 24hrs or 1+23hrs. Differences between treatments were highly significant (2-way ANOVA, Tukey’s multiple comparison, p<0.0001). n=9-11 fish, 4 NMs/fish for each condition. B) The rate of delayed hair cell loss is dependent on initial G418 concentration. Fish were treated with doses of G418 (25, 50, 100, 200 µM) for 1hr, then rinsed and incubated in fresh medium. Loss of hair cells was assessed at 5hrs, 11hrs, 17hrs and 23hrs after the 1hr incubation period. Increasing initial dose results in more rapid delayed hair cell loss. Differences between 25 µM, 50 µM and 200 µM treatments were significant (Tukey’s multiple comparison, p<0.0001). There was no significant difference between 100 µM and 200 µm treatments. n=8-13 fish, 4 NMs/fish for each treatment. Error bars represent Standard Deviation.

**Supplemental Figure 2.** Different cytoplasmic calcium responses during acute or delayed hair cell death. Fluorescence changes above baseline (F/F_0_) from cytoRGECO in response to AG addition, imaged by spinning disk microscopy at 30 sec intervals. Individual traces represent responses of individual cells. Traces are aligned to time of cell fragmentation. A) Changes in cytoRGECO signal in cells undergoing acute death in response to 100 µM neomycin. Hair cells were imaged during the first hour of neomycin exposure. Increases in mitochondrial Ca^2+^ were observed in 10/10 dying cells. B) Changes in cytoRGECO signal in cells undergoing delayed death after exposure to 100 µM G418. Cells were exposed to G418 for 1hr, followed by rinses and incubation in fresh embryo medium (EM) for 1.5h, and then imaged over an additional 2hr period. Increases in cytoplasmic Ca^2+^ were observed in 2/16 dying cells. C) Changes in cytoRGECO signal in cells undergoing acute death in response to 400 µM G418. Hair cells were imaged during the first hour of G418 exposure. Increases in cytoplasmic Ca^2+^ were observed in 4/12 dying cells. D) Maximum cytoRGECO signal compared to baseline for dying cells after neomycin or G418 exposure. *** Kruskal-Wallis with Dunn’s multiple comparison test p<0.0005. Error bars represent Standard Deviation.

**Supplemental Figure 3.** The mitochondrially-targeted antioxidant mitoTEMPO protects against high-dose acute G418 exposure. 50 µM mitoTEMPO was added for 30min before AG, co-treated with G418 for 1hr. Little damage is seen after 400µM G418 treatment with or without mitoTEMPO. Treatment with mitoTEMPO protects against 800µM G418. **** Two-way ANOVA, Sidak’s multiple comparison, p<0.0001. ns p = 0.73. n=9-11 fish, 4 NMs/fish for each treatment group. Error bars represent Standard Deviation.

**Supplemental Figure 4.** Effects of mitoTEMPO on G418-TR and Neo-TR uptake. 50 µM mitoTEMPO treatment results in an increase in G418 uptake. ***, Mann Whitney, 0.0002. B) 50 µM mitoTEMPO does not alter Neo-TR uptake. ns Mann Whitney, p=0.43 n=12 fish, 6-7 NM/fish in each treatment group. Shading indicates measurements from two separate experiments. Error bars represent Standard Deviation.

**Supplemental Figure 5.** Texas Red label does not change efficacy of aminoglycosides. A) Comparison of G418 to G418-TR. There are no differences in dose-response relationships for either 1hr or 1+23hr treatments (Two-way ANOVA, Sidak’s multiple comparison test). B) Comparison of neomycin to Neo-TR. There is no difference between dose-response relationships between unlabeled and labeled neomycin (Two-way ANOVA, Sidak’s multiple comparison test).

**Supplemental Figure 6.** Automated segmentation of Rab7-labeled vesicles and neuromasts. A) Masks generated for vesicles, neuromasts, cytoplasm (neuromast-vesicle) and background. B) Ratio of vesicle area to neuromast area. There is no difference between drug treatment conditions (unpaired T test). C, D) Mean fluorescence values for whole neuromast (C) or vesicles (D). Error bars represent Standard Deviation.

**Supplemental Figure 7.** Bafilomycin A1 protects hair cells against G418 but not neomycin.

A) 100 nM Bafilomycin A1 treatment offers robust protection against gentamicin (Two-way ANOVA, Sidak’s multiple comparison: ns, 10 µM; p<0.0001, 25, 50, 100 µM; p<0.01, 200 µM). B) Bafilomycin A1 does not protect against neomycin at any concentration. n=9-11 fish, 4 NMs/fish for each treatment group. Error bars represent Standard Deviation.

**Supplemental Figure 8.** Effects of GPN and Bafilomycin A1 on G418-TR uptake. A) 250 µM GPN treatment does not change G418 uptake. ns, Mann Whitney. B) 100 nM Bafilomycin A1 significantly reduces uptake. **** Mann Whitney, p<0.0001. n=12 fish, 6-7 NM/fish in each treatment group. Error bars represent Standard Deviation.

Supplemental Table 1. Tukey’s posthoc comparisons of values from 2-way ANOVA of neomycin and gentamicin dose-response functions in Figure 1D, E.

|  | Neomycin | | | Gentamicin | | |
| --- | --- | --- | --- | --- | --- | --- |
| AG Conc. (µM) | 1h vs 24h | 1h vs 1+23h | 24h vs 1+23h | 1h vs 24h | 1h vs 1+23h | 24h vs 1+23h |
| 0 | ns | ns | ns | ns | ns | ns |
| 1 | ns | ns | ns | <0.0001 | 0.05 | <0.0001 |
| 10 | <0.0001 | <0.0001 | ns | <0.0001 | <0.0001 | <0.0001 |
| 25 | <0.0001 | 0.0031 | <0.0001 | <0.0001 | <0.0001 | <0.0001 |
| 50 | 0.01 | <0.0001 | <0.0001 | <0.0001 | <0.0001 | <0.0001 |
| 100 | ns | <0.0001 | <0.0001 | <0.0001 | <0.0001 | <0.0001 |
| 200 | ns | <0.0001 | 0.0002 | <0.0001 | <0.0001 | <0.0001 |
| 400 |  |  |  | <0.0001 | <0.0001 | <0.0001 |

Supplemental Table 2. Šídák's multiple comparisons test of values from 2-way ANOVA of neomycin and gentamicin dose-response functions in Figure 1D, E. Values for each dose compared to no AG addition.

|  | Neomycin | | | Gentamicin | | |
| --- | --- | --- | --- | --- | --- | --- |
| AG Conc. (µM) | 1h | 24h | 1+23h | 1h | 24h | 1+23h |
| 1 | ns | ns | ns | ns | <0.0001 | ns |
| 10 | ns | <0.0001 | 0.0002 | ns | <0.0001 | <0.0001 |
| 25 | <0.0001 | <0.0001 | 0.0014 | ns | <0.0001 | <0.0001 |
| 50 | <0.0001 | <0.0001 | <0.0001 | ns | <0.0001 | <0.0001 |
| 100 | <0.0001 | <0.0001 | <0.0001 | ns | <0.0001 | <0.0001 |
| 200 | <0.0001 | <0.0001 | <0.0001 | 0.0017 | <0.0001 | <0.0001 |
| 400 |  |  |  | <0.0001 | <0.0001 | <0.0001 |
